# Wie werden die KRINKO-Empfehlungen im Öffentlichen Gesundheitsdienst (ÖGD) wahrgenommen? Bericht über die Ergebnisse einer Onlinebefragung von Mitarbeitenden des ÖGD durch das Robert Koch-Institut (RKI)

**DOI:** 10.1007/s00103-025-04131-4

**Published:** 2025-10-21

**Authors:** Melanie M. P. Winkler, Franziska Lexow, Esther E. Dirks, Melanie Brunke, Yari Osenberg, Mardjan Arvand

**Affiliations:** 1 Stabsstelle Akademisierte Gesundheitsfachberufe, Charité – Universitätsmedizin Berlin, Berlin, Deutschland; 2 Abteilung 1 Infektionskrankheiten, FG 14 Angewandte Infektions- u. Krankenhaushygiene, Robert Koch-Institut, Nordufer 20, 13353 Berlin, Deutschland; 3 Zentrum für Biologische Gefahren und Spezielle Pathogene, ZBS 7 Strategie und Einsatz, Robert Koch-Institut, Berlin, Deutschland; 4 MFI Methodenentwicklung, Forschungsinfrastruktur und Informationstechnologie, MF 2 Fachdaten-Kompetenzzentrum, Robert Koch-Institut, Berlin, Deutschland; 5 Robert Koch-Institut, Berlin, Deutschland

## Hintergrund

Seit nunmehr 5 Jahrzehnten erstellt die Kommission für Infektionsprävention in medizinischen Einrichtungen und in Einrichtungen und Unternehmen der Pflege und Eingliederungshilfe (ehemals Kommission für Krankenhaushygiene und Infektionsprävention) (KRINKO) beim Robert Koch-Institut (RKI) unter Berücksichtigung aktueller infektionsepidemiologischer Erkenntnisse Empfehlungen zur Prävention nosokomialer und weiterer Infektionen sowie zu betrieblich-organisatorischen und baulich-funktionellen Maßnahmen der Hygiene in Einrichtungen des Gesundheitswesens (alle aktuellen Empfehlungen der KRINKO verfügbar unter www.rki.de/krinko-empfehlungen).

Die Empfehlungen der KRINKO bilden gemäß § 23 Infektionsschutzgesetz (IfSG) [1] national die Basis für Maßnahmen zur Infektionsprävention und -kontrolle im Gesundheitswesen in Deutschland. Gleichzeitig dienen sie als Orientierungs- und Verständigungsgrundlage zwischen den Mitarbeitenden in medizinischen Einrichtungen und dem Öffentlichen Gesundheitsdienst (ÖGD).

Der ÖGD in Deutschland umfasst verschiedene Einrichtungen und Institute auf kommunaler sowie auf Länder- und Bundesebene. Das Gesundheitsamt (GA, im Plural GÄ) ist als untere Gesundheitsbehörde auf kommunaler Ebene (KE) tätig. Auf Länderebene (LE) sind die Landesgesundheitsministerien sowie Landesämter und -institute tätig. Auf Bundesebene (BE) setzt sich der ÖGD u. a. aus dem Bundesministerium für Gesundheit (BMG) und Bundesoberbehörden zusammen. Zu Letzteren gehören beispielsweise das RKI, das Bundesinstitut für Öffentliche Gesundheit (BIÖG; ehemals Bundeszentrale für gesundheitliche Aufklärung, BZgA) oder das Bundesinstitut für Arzneimittel und Medizinprodukte (BfArM; [2]).

Ende 2024 waren 26.655 Personen in den 377 GÄ in Deutschland tätig [3]. Der ÖGD, insbesondere die Mitarbeitenden in den GÄ, die maßgeblich mit der Beratung zu den Empfehlungen sowie der Überwachung ihrer Umsetzung betraut sind, stellt somit eine große und wichtige Zielgruppe für die KRINKO-Empfehlungen dar.

Obwohl der ÖGD bereits langjährige Erfahrung mit der Überwachung der Implementierung der KRINKO-Empfehlungen aufweist, gab es bisher noch keine Erkenntnisse darüber, inwieweit diese Empfehlungen im beruflichen Alltag des ÖGD genutzt, nachvollzogen und als hilfreich sowie verständlich wahrgenommen werden („Perzeption“). Ebenso war unklar, über welche Kommunikationskanäle und in welcher Form die KRINKO-Empfehlungen den ÖGD erreichen.

Bereits im Jahr 2022 initiierte das Fachgebiet (FG) 14 „Angewandte Infektions- und Krankenhaushygiene“ des RKI eine Pilotbefragung zur Untersuchung der „Perzeption der KRINKO-Empfehlungen in der Fachöffentlichkeit“ [4]. Diese Befragung richtete sich an das Hygienefachpersonal, das sich im Berufsalltag maßgeblich mit Aspekten der Infektionsprävention in Einrichtungen des Gesundheitswesens befasst.

Um den Wissensbedarf auch für den ÖGD zu adressieren, führte das FG 14, in dem auch die Geschäftsstelle und das wissenschaftliche Sekretariat der KRINKO angesiedelt sind, im Rahmen des RKI-Projekts „Public Health Impact-Analysen im Verbund mit Public Health-Akteuren“ im Jahr 2023 eine Onlinebefragung der Mitarbeitenden im ÖGD durch. In diesem Artikel wird über die Ergebnisse der ÖGD-Befragung und die gewonnenen Erkenntnisse berichtet.

## Konzept und Durchführung

Für die Befragung wurde ein Fragebogen entworfen (s. Onlinematerial 1), dessen Struktur vergleichbar mit jener aus der Pilotbefragung ist (vgl. Anhang in [4]). Der Fragebogen wurde im Rahmen eines Online-Workshops mit 7 Vertreterinnen und Vertretern des ÖGD und der KRINKO diskutiert und abgestimmt.

Um eine hohe Rücklaufquote und niederschwellige Teilnahme zu erzielen, wurde für das Ausfüllen des Fragebogens eine Bearbeitungszeit von ca. 10 min eingeplant. Zunächst erfolgte eine Prüfung des Fragebogens auf datenschutzrechtliche Vorgaben hinsichtlich Sicherstellung der Anonymität der zu gewinnenden Datensätze, im Anschluss wurde der Fragebogen mit der Online-Survey-Software VOXCO (VOXCO, Montreal, Kanada) mit Unterstützung des Epidemiologischen Daten- und Befragungszentrums (FG 21) programmiert. Für die darauffolgende Extraktion und Aufbereitung der Daten sowie die Erstellung der Abbildungen wurde die Programmiersprache R (Version 4.2.1) verwendet. Die im Text angegebenen relativen Häufigkeiten (Prozentwerte) werden immer in Bezug auf die Anzahl der jeweils antwortenden Personen aus den entsprechenden Einrichtungsebenen (KE, LE) gebildet. In den Abbildungen werden die absoluten Häufigkeiten dargestellt. Die Datenverarbeitung fand in Zusammenarbeit mit dem MF 2 Fachdaten-Kompetenzzentrum der Abteilung Methodenentwicklung, Forschungsinfrastruktur und Informationstechnologie (MFI) des RKI statt.

Der Fragebogen beinhaltete 17 Fragen und wurde thematisch in 3 Blöcke gegliedert. In der Einstiegsfrage wurde eine Filterfunktion integriert, um sicherzustellen, dass nur Teilnehmende, die die Zugehörigkeit zum ÖGD bejahten, den weiteren Fragebogen ausfüllen konnten. Nach Beantwortung dieser ersten Frage bestand zu jeder Zeit die Möglichkeit, einzelne Fragen auszulassen. Dementsprechend variiert die Anzahl der Antworten bei den jeweiligen Fragen. Der Großteil der Fragen wurde im Multiple-Choice-Format konzipiert, bei dem je nach Frage eine oder mehrere Antwortmöglichkeiten gewählt werden konnten. Bei einer Frage wurde mithilfe einer Likert-Skala um eine Einschätzung gebeten.

Der erste Block des Fragebogens befasste sich mit den Charakteristika der Teilnehmenden. Hierbei wurden der berufliche Hintergrund, die Art der Behörde bzw. Einrichtung, in denen die Teilnehmenden tätig sind, sowie das Bundesland, in dem sie zur Zeit der Befragung tätig waren, abgefragt. Im zweiten Block standen die Wahrnehmung der KRINKO, ihrer Arbeitsweise und ihrer Empfehlungen im Mittelpunkt. Der dritte Block des Fragebogens bezog sich spezifisch auf das Empfehlungsdokument „Anforderungen an die Hygiene bei der Reinigung und Desinfektion von Flächen“ [5] sowie auf dessen „informativen Anhang“ [6].

Zur Bekanntmachung der Onlinebefragung wurden z. B. Fachzeitschriften und RKI-assoziierte und externe digitale Formate genutzt.

## Ergebnisse aus der Befragung

Die Online-Befragung fand vom 01.11.2023 bis zum 15.12.2023 statt. Insgesamt wurde der Fragebogen 465-mal vollständig ausgefüllt, 95-mal wurden nicht alle Fragen beantwortet.

### Block I: Allgemeine Hintergrundinformationen (Charakteristika der Befragten)

Die überwiegende Mehrheit der Teilnehmenden, insgesamt 497 Personen, gab an, im GA tätig zu sein, also auf KE (496 Personen haben hier präzise Angaben zu ihrer jeweiligen Funktion gemacht). Auf LE beteiligten sich insgesamt 51 Personen an der Befragung. 12 Teilnehmende gaben eine Tätigkeit auf BE an. Aufgrund der geringen Zahl der Teilnehmenden auf BE und der daraus resultierenden mangelnden Repräsentativität konnten die Antworten der Befragten auf BE in diesem Bericht nicht ausgewertet werden.

**Abb. 1:**
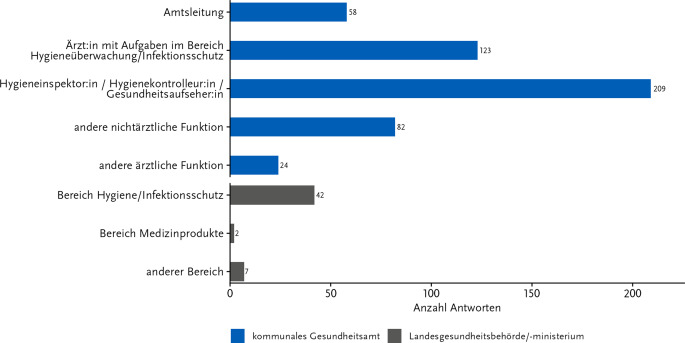
Art der Behörde/Funktion der Teilnehmenden in ihrer jeweiligen Behörde. Antworten auf die Frage: „Ich bin tätig in einer/einem …, speziell in der Funktion …“ Anzahl Antworten: 547; davon Gesundheitsamt: 496 und Länderebene: 51. Onlinebefragung zur Wahrnehmung der KRINKO-Empfehlungen im ÖGD 11-12/2023

Aus den GÄ gaben 42,1 % der Teilnehmenden an, als Hygieneinspektor:in/Hygienekontrolleur:in/Gesundheitsaufseher:in tätig zu sein, 24,8 % als Ärzt:in mit Aufgaben im Bereich Hygieneüberwachung/Infektionsschutz, 16,5 % in anderer nichtärztlicher Funktion und 4,8 % in anderer ärztlicher Funktion. Die Amtsleitung hatten 11,7 % der Personen inne (Abb. 1). Bei den auf LE-Tätigen gaben 82,4 % den Bereich Hygiene/Infektionsschutz als Arbeitsfeld an, 3,9 % den Bereich Medizinprodukte und 13,7 % andere Bereiche.

**Abb. 2:**
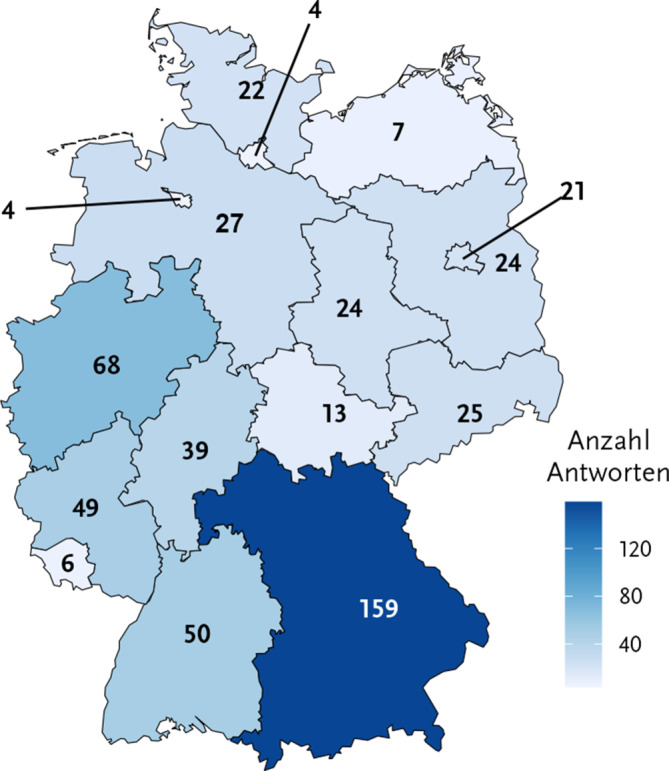
Geografische Verteilung des Tätigkeitsorts der Teilnehmenden durch Angabe des Bundeslands. Gesamtzahl der Antworten: 542. Onlinebefragung zur Wahrnehmung der KRINKO-Empfehlungen im ÖGD 11-12/2023

Die Befragung erreichte Personen aus allen Bundesländern (Abb. 2). Bevölkerungsreiche Bundesländer waren stärker vertreten, die wenigsten Rückmeldungen kamen aus den Stadtstaaten Hamburg und Bremen sowie aus den Bundesländern Saarland und Mecklenburg-Vorpommern.

**Abb. 3:**
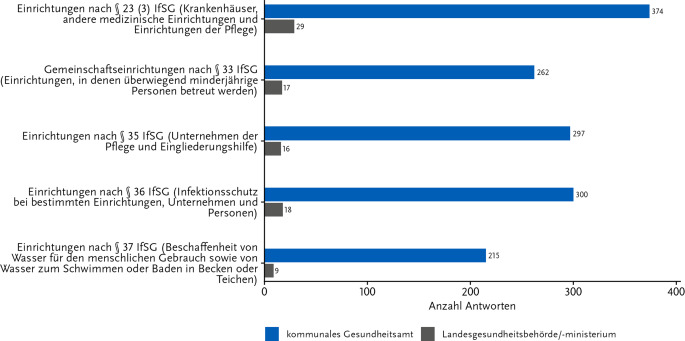
Arbeitsschwerpunkte der Teilnehmenden, die Beratungs- und Überwachungsaufgaben im Bereich der Hygiene erfüllen. Antworten auf die Frage: „Welche Beratungs- und Überwachungsaufgaben im Bereich der Hygiene sind das genau?“ Anzahl Antworten: 446; davon Gesundheitsamt: 417 und Länderebene: 29. Mehrfachnennungen waren möglich. Diese Frage war nur an Teilnehmende gerichtet, die in der vorherigen Frage „Beratungs- und Überwachungsaufgaben im Bereich der Hygiene“ angegeben hatten. Onlinebefragung zur Wahrnehmung der KRINKO-Empfehlungen im ÖGD 11-12/2023

Bei der Frage zum Schwerpunkt der beruflichen Tätigkeiten lag in beiden Gruppen der Fokus vorrangig auf Beratungs- und Überwachungsaufgaben in der Hygiene (86,7 % auf KE; 64,4 % auf LE; s. Onlinematerial 2, Abb. A1). Um den Arbeitsschwerpunkt dieser Befragten noch näher zu erfassen, wurden die Befragten anschließend gebeten, die Art der gemäß IfSG definierten Einrichtungen anzugeben, für die sie Beratungs- und Überwachungsaufgaben ausführten (Abb. 3). Die überwiegende Mehrheit der GA-Mitarbeitenden (89,9 %) und alle LE-Tätigen (100,0 %) waren mit Aufgaben in Einrichtungen entsprechend § 23 (3) IfSG, also in Krankenhäusern und anderen medizinischen Einrichtungen, betraut.

### Block II: Perzeption der KRINKO und der KRINKO-Empfehlungen allgemein

Der zweite Block des Fragebogens begann mit einer Selbsteinschätzung darüber, wie vertraut sich Antwortende mit den für die eigene Tätigkeit als relevant erachteten KRINKO-Empfehlungen fühlten – auf einer Likert-Skala von 0 („gar nicht“) bis 10 („sehr gut“), welche sowohl auf KE als auch auf LE tendenziell in Richtung „gut/sehr gut“ eingeschätzt wurde (s. Onlinematerial 2, Abb. A2).

Um zu untersuchen, wie nützlich die Empfehlungen im Berufsalltag der Zielgruppen empfunden werden, ist ein relevanter Faktor die Häufigkeit, mit der die Befragten in den originalen Empfehlungsdokumenten nachlesen. Dabei führten 39,8 % der Antwortenden aus GÄ an, anlassbezogen in den Originaltexten der KRINKO-Empfehlungen nachzulesen, während die Mehrheit der LE-Mitarbeitenden äußerte, mehrmals im Monat die Originaltexte zu Rate zu ziehen (35,7 %; s. Onlinematerial 2, Abb. A3). Dabei gab etwa jede 5. Person der GA-Mitarbeitenden (19,8 %) sowie jede 6. Person auf LE (16,7 %) an, nur unregelmäßig in den Texten nachzulesen.

**Abb. 4:**
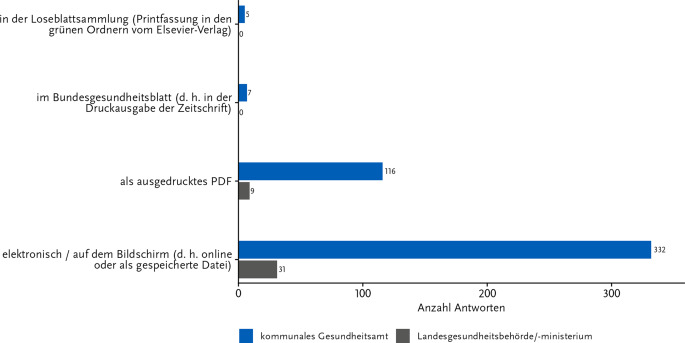
Bevorzugte Rezipiens der KRINKO-Empfehlungen. Antworten auf die Frage: „In welcher Form lesen Sie die Originaltexte der Empfehlungen bevorzugt?“ Anzahl Antworten: 500; davon Gesundheitsamt: 460 und Länderebene: 40. Diese Frage war nur an Teilnehmende gerichtet, die in der vorherigen Frage nicht mit „nie“ geantwortet hatten. Onlinebefragung zur Wahrnehmung der KRINKO-Empfehlungen im ÖGD 11-12/2023

Die Empfehlungsdokumente der KRINKO sind in verschiedenen Formen verfügbar und unterscheiden sich dabei teilweise im Layout. 3 Viertel der Befragten (72,2 % der GA-Tätigen; 77,5 % der auf LE-Tätigen) bevorzugten die elektronische Form der Texte (d. h. online oder als gespeicherte Datei; Abb. 4). Etwa jede 4. Person präferierte ausgedruckte PDF-Dateien (25,2 % GA; 22,5 % LE). Lediglich 1,1 % bzw. 1,5 % der Teilnehmenden aus den GÄ nutzten die Empfehlungsdokumente in Form der Loseblattsammlung oder der Printausgabe des Bundesgesundheitsblatts (für Erläuterungen dieser Formen s. Pilotbericht 2022 [4]).

Sobald die KRINKO neue Empfehlungen veröffentlicht, wird dies über verschiedene Kanäle kommuniziert. Bei der Frage, auf welchen Wegen die Befragten über neue Empfehlungen der KRINKO erfahren, wurde in beiden Gruppen die Homepage des RKI am häufigsten angegeben, gefolgt von dem Austausch mit dem Kollegium sowie der Information über den RKI-Newsletter (Tab. 1).

**Tab. 1:** Wahrnehmungswege neuer KRINKO-Empfehlungen. Antworten auf die Frage: „Auf welchen Wegen erfahren Sie von neuen KRINKO-Empfehlungen?“ Mehrfachnennungen waren möglich. Onlinebefragung zur Wahrnehmung der KRINKO-Empfehlungen im ÖGD 11-12/2023

Antworten insgesamt: 501	GA (*n* = 460; %)	LE (*n* = 41; %)
Bundesgesundheitsblatt	30,7	26,8
Homepage des RKI	68,7	63,4
RKI-Newsletter	48,0	53,7
Social-Media-Kanäle des RKI (z. B. X, LinkedIn)	1,5	2,4
Andere Social-Media-Kanäle	1,5	0,0
Agora (Plattform für Kommunikation und Kollaboration im ÖGD)	3,5	0,0
ÖGD-News-App der AÖGW	17,4	0,0
Informationen der Fachgesellschaften	13,0	26,8
Intranet meiner Einrichtung	5,0	4,9
Fachzeitschriften	19,6	14,6
Kongresse, z. B. Vorträge	25,7	34,2
Kolleg:innen	53,5	51,2
Andere	13,0	7,3

*AÖGW* Akademie für Öffentliches Gesundheitswesen, *ÖGD* Öffentlicher Gesundheitsdienst, *RKI* Robert Koch-Institut

Bei der Frage, woher die Befragten die Inhalte der KRINKO-Empfehlungen beziehen (s. Onlinematerial 2, Tab. A1), ließ sich eine tätigkeitsabhängige Unterscheidung erkennen. Sowohl die Mitarbeitenden auf KE als auch auf LE gaben in der Mehrheit an, die Originaltexte der Empfehlungen selbst als Informationsquelle zu nutzen.

Das Verständnis der Empfehlungen ist notwendig, um diese auch adäquat vermitteln zu können, da dies eine wesentliche Aufgabe im ÖGD darstellt. Bei Verständnisfragen bezüglich der KRINKO-Empfehlungen (s. Onlinematerial 2, Tab. A2) präferierten beide Gruppen den Austausch mit dem Kollegium innerhalb der eigenen Einrichtung (80,7 % GA; 75,6 % LE), gefolgt von der Recherche explizit auf der RKI-Homepage (65,4 % GA; 51,2 % LE) bzw. einer freien Internetsuche (58,0 % GA; 53,7 % LE). Auf LE wandte sich jede 5. Person an das Infektionshygiene-Postfach des RKI (19,5 %), wohingegen nur 4,4 % der GA-Mitarbeitenden diese Option in Anspruch nahmen.

Bei der Frage, ob die Teilnehmenden die Inhalte der KRINKO-Empfehlungen auch an andere vermitteln, bejahten dies 63,4 % auf LE, während die Mehrheit der Mitarbeitenden aus dem GA (48,6 %) angab, tätigkeitsbedingt die Inhalte eher indirekt zu kommunizieren (s. Onlinematerial 2, Abb. A4). Dabei wurde deutlich, dass die Weitergabe der Empfehlungsinhalte bei den GA-Mitarbeitenden vor allem bei Begehungen bzw. in Begehungsberichten und in individuellen Beratungen erfolgte. LE-Mitarbeitende vermitteln die Inhalte am häufigsten in individuellen Beratungen oder Gesprächen bei Begehungen, aber auch in Form von Vorträgen (54,8 %) und selbsterstellten Informationsmaterialien (41,9 %; s. Onlinematerial 2, Tab. A3).

Für die Vermittlung der Inhalte der KRINKO-Empfehlungen wurden vor allem die Originaltexte genutzt, um z. B. Auszüge daraus darzustellen (s. Onlinematerial 2, Tab. A4). Hervorzuheben ist hier der deutliche Unterschied in der Nutzung der Musterpräsentationen des RKI. Während 41,9 % der LE-Beschäftigten diese nutzten, taten dies nur 29,4 % der GA-Mitarbeitenden.

**Abb. 5:**
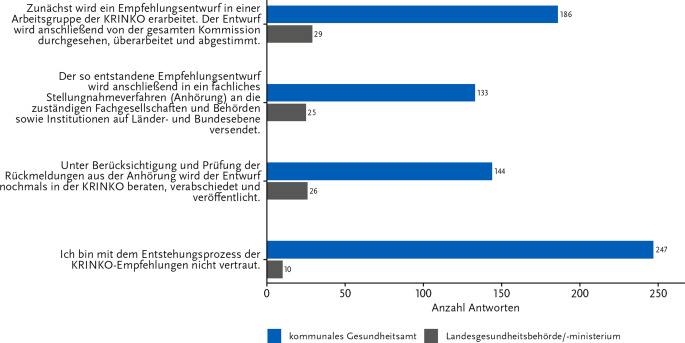
Kenntnisse zum Entstehungsprozess der KRINKO-Empfehlungen. Antworten auf die Frage: „Welche Aspekte aus dem Entstehungsprozess einer KRINKO-Empfehlung sind Ihnen bekannt?“ Anzahl Antworten: 484; davon Gesundheitsamt: 444 und Länderebene: 40. Mehrfachnennungen waren möglich. Onlinebefragung zur Wahrnehmung der KRINKO-Empfehlungen im ÖGD 11-12/2023

Um einen Einblick in die Kenntnisse der Teilnehmenden über die Arbeitsweise der KRINKO zu erhalten, wurde der Entstehungsprozess der Empfehlungen in Frage 11 in 3 Schritte unterteilt und betrachtet (Abb. 5; kongruent zur Pilotbefragung [4]). Von besonderem Interesse war die Bekanntheit des öffentlichen Stellungnahmeverfahrens, um zu sehen, wie verbreitet das Wissen über die Involvierung von Teilen des ÖGD bei der Erstellung der KRINKO-Empfehlungen ist. Hierbei zeigten sich deutlichere Unterschiede in den Antworthäufigkeiten zwischen den beiden Gruppen.

Die Mehrheit der auf LE Teilnehmenden zeigte sich vertraut mit (Teilen) der Entstehung einer KRINKO-Empfehlung, wohingegen mehr als der Hälfte der antwortenden GA-Mitarbeitenden laut eigener Aussage der Entstehungsprozess nicht bekannt war (55,6 % GA; 25,0 % LE). Die Vertrautheit mit dem Stellungnahmeverfahren war in beiden Gruppen geringfügig niedriger ausgeprägt als die anderen Aspekte des Entstehungsprozesses.

**Abb. 6:**
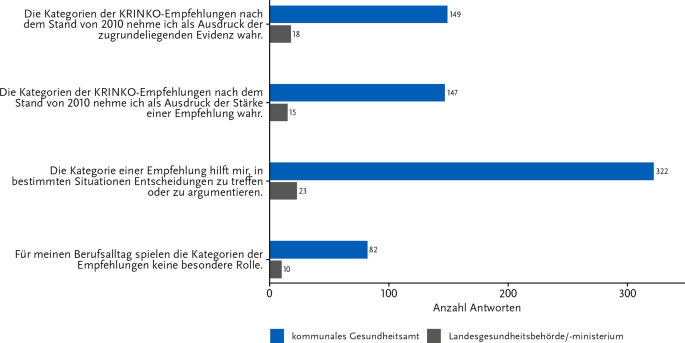
Kenntnisse zu den Evidenzkategorien innerhalb der KRINKO-Empfehlungen. Antworten auf die Frage: „Welche Aussagen treffen für Sie in Bezug auf die Kategorien der KRINKO-Empfehlungen (nach dem Stand von 2010) zu?“ Anzahl Antworten: 475; davon Gesundheitsamt: 435 und Länderebene: 40. Mehrfachnennungen waren möglich. Onlinebefragung zur Wahrnehmung der KRINKO-Empfehlungen im ÖGD 11-12/2023

Eine ebenso zentrale Rolle bei der Wahrnehmung der KRINKO-Empfehlungen spielen die Kategorien, mit denen die Qualität der wissenschaftlichen Evidenz für die jeweiligen Einzelempfehlungen angegeben wird. Eine konkrete „Empfehlungsstärke“ ist daraus nicht abzuleiten (für ausführliche Erläuterung s. [7]). Bei der diesbezüglichen Frage wurde jedoch häufig angegeben, dass die Kategorien als Indikator sowohl für die Qualität der Evidenz (34,3 % GA; 45,0 % LE) als auch für die Stärke einer Empfehlung (33,8 % GA; 37,5 % LE) wahrgenommen werden (Abb. 6). Ein Fünftel der GA-Mitarbeitenden (18,9 %) sowie ein Viertel der LE-Mitarbeitenden (25,0 %) äußerten, dass die Kategorien für ihren Berufsalltag nicht von besonderer Relevanz wären. Für die Mehrheit der Befragten stellten die Kategorien jedoch eine deutliche Unterstützung bei der Entscheidungsfindung bzw. Argumentation dar (74,0 % GA; 57,5 % LE).

### Block III: Perzeption des Empfehlungsdokuments „Anforderungen an die Hygiene bei der Reinigung und Desinfektion von Flächen“

Der dritte Block des Fragebogens legte den Fokus auf die Perzeption des Empfehlungsdokuments „Anforderungen an die Hygiene bei der Reinigung und Desinfektion von Flächen“ [5], welches zum Zeitpunkt der Befragung seit einem Jahr veröffentlicht war. Ein großer Vorteil dieser Empfehlung ist ihre Relevanz für jede Art medizinischer Einrichtungen. Damit bot sich die Möglichkeit, ein breites Spektrum an Befragten zu adressieren. Hervorzuheben ist hierbei auch dessen „informativer Anhang“ [6], der weiterführende Informationen und eine zusätzliche theoretische Orientierung ohne Empfehlungscharakter bzw. Vermutungswirkung zur Verfügung stellt (für Erläuterungen zur Vermutungswirkung s. Pilotbericht 2022 [4]). Bis dahin hatte die KRINKO diese Form der Publikation selten genutzt.

Der dritte Block begann mit der Frage zur Bekanntheit des Empfehlungsdokuments. In beiden Gruppen der Befragten gab jeweils die Hälfte an, mit dem Empfehlungsdokument *teilweise* inhaltlich vertraut zu sein (52,9 % der GA-Tätigen; 52,5 % der Beteiligten auf LE; s. Onlinematerial 2, Abb. A5). Einem Zehntel der Befragten war die Empfehlung zum Zeitpunkt der Befragung inhaltlich nicht bekannt (9,4 % GA; 10,0 % LE). Hinsichtlich der Nutzung des Originaltextes dieser Empfehlung zeigte sich, dass dieser vorwiegend in besonderen Situationen, bei Fragen oder Problemen zur Hand genommen wird (69,4 % GA; 70,0 % LE; s. Onlinematerial 2, Abb. A6). Circa ein Viertel der GA-Mitarbeitenden äußerte hierbei, im Berufsalltag regelmäßig in dieser Empfehlung nachzulesen (26,7 %).

Im Folgenden wurde in 2 weiteren Fragen um eine Einschätzung der Verständlichkeit der Sprache und der Nachvollziehbarkeit des Aufbaus der Empfehlung gebeten. Dabei schätzte etwas mehr als die Hälfte der Befragten die Sprache und Formulierungen als relativ verständlich (jeweils 51,3 %) und den Aufbau und die Struktur des Dokuments als einigermaßen nachvollziehbar (57,7 % GA; 51,3 % LE) ein (s. Onlinematerial 2, Abb. A7 und Abb. A8). Ein Zehntel empfand die Sprache als zu kompliziert (11,7 % GA; 10,3 % LE), nur Wenige schätzten den Aufbau der Empfehlung als schwer nachvollziehbar ein (6,2 % GA; 7,7 % LE).

Der informative Anhang der Empfehlung war zu dem Zeitraum der Befragung etwa der Hälfte der Teilnehmenden bekannt (48,4 % GA; 56,4 % LE; s. Onlinematerial 2, Abb. A9). Für jede fünfte (20,5 %) auf LE tätige Person war dieser auch relevant für den Berufsalltag, dies traf ebenso auf 13,9 % der GA-Mitarbeitenden zu.

Zur umfassenden Analyse der Perzeption der KRINKO-Empfehlungen gehört ebenfalls die Identifikation geeigneter Kommunikationskanäle, um gezielt und direkt die relevanten Adressatengruppen über KRINKO-spezifische Aspekte zu informieren. Daher konnten die Teilnehmenden abschließend angeben, über welchen Weg sie von dieser Befragung erfahren hatten. Dabei wurde deutlich, dass die Befragten vorrangig durch das Kollegium auf diese Befragung hingewiesen wurden (37,1 % GA; 43,6 % LE; s. Onlinematerial 2, Tab. A5). Außerdem spielte der Internetauftritt des RKI eine wesentliche Rolle. Hier wurden sowohl Anzeigen im *Epidemiologischen Bulletin* als auch die Homepage selbst benannt. Die Ankündigungen über die anderen digitalen Formate trugen kaum zur Bekanntmachung dieser Befragung bei.

## Erkenntnisse zur Nutzung und Perzeption der KRINKO-Empfehlungen

### Einsatz der KRINKO-Empfehlungen im ÖGD

Organisationsformen und Zuständigkeitsbereiche der Behörden des ÖGD sind in Deutschland aufgrund der föderalen Strukturen sehr heterogen. Entsprechend ihren Zuständigkeiten und Aufgaben nutzen die verschiedenen Gruppen die KRINKO-Empfehlungen für unterschiedliche Zwecke, etwa auch für Schulungen unterschiedlicher Zielgruppen. Auf der LE werden sie vermutlich zur Erstellung landesspezifischer Rechtsverordnungen für infektionshygienische Regelungen (z. B. in Landeshygieneverordnungen) oder zur Überprüfung der Fachaufsichtspflichten kommunaler Behörden herangezogen, während sie auf KE, also im Bereich der GÄ, häufig im Rahmen von Beratungen oder Hygienebegehungen medizinischer Einrichtungen Anwendung finden.

Um Unterschiede in der Perzeption feiner darstellen und ggf. angepasste Optimierungsstrategien, wie etwa für Kommunikationswege, abzuleiten, wurde in der vorliegenden Befragung zwischen den Gruppen auf BE, LE und KE differenziert. Die BE konnte aufgrund der geringen Teilnehmerzahl nicht ausgewertet werden. Die Antwortverteilung auf KE und LE war bei vielen der gestellten Fragen ähnlich. Erhebliche grundsätzliche Unterschiede zwischen den beiden Gruppen waren nicht erkennbar. Sie zeigten sich nur in kleineren Teilbereichen. Dies dürfte größtenteils auf die Interaktion mit unterschiedlichen Zielgruppen im Rahmen der jeweiligen Zuständigkeiten zurückzuführen sein. Möglicherweise führt die kleinere Stichprobe auf der LE zu statistischen Ungenauigkeiten beim Vergleich beider Gruppen.

Prinzipiell unterstreicht die hohe Beteiligung an der aktuellen Befragung und der vorangegangene Wunsch aus Reihen des ÖGD, diesen als Zielgruppe in die Befragung einzubeziehen, die Relevanz der KRINKO-Empfehlungen im beruflichen Alltag der ÖGD-Mitarbeitenden. Der Anteil der Teilnehmenden aus GÄ war mit 90,7 % besonders hoch, hier werden KRINKO-Empfehlungen am häufigsten im Rahmen der direkten Beratungs- und Überwachungsfunktion genutzt.

### Formate und Zugriffsstrategien für KRINKO-Empfehlungen

Es wurde deutlich, dass die Nutzung der Empfehlungsdokumente wie auch laut der Befragung des Hygienefachpersonals 2022 überwiegend in elektronischer Form erfolgt (online oder als PDF), wobei ein Viertel der Befragten die PDF-Dokumente in ausgedruckter Form für den Arbeitsalltag nutzt. Ein großer Vorteil bei der Nutzung digitaler Dokumente liegt hierbei wahrscheinlich in der Möglichkeit der automatisierten Stichwortsuche über Tastenkombinationen, um relevante Aspekte leichter zu finden. Dafür spricht auch, dass die Befragten angaben, die Empfehlungen überwiegend anlassbezogen zu Rate zu ziehen. Die digitale Stichwortsuche unterstützt hierbei, effizient auf spezifische Inhalte zuzugreifen und diese z. B. bei Vor-Ort-Begehungen heranzuziehen und bestimmte Entscheidungen zu begründen. Diese Ergebnisse decken sich mit denen aus der Pilotbefragung [4]. Um sich Inhalte aus KRINKO-Empfehlungen anzueignen, nutzen insbesondere Mitarbeitende der GÄ neben den Originaldokumenten auch verstärkt interne und externe Fortbildungen, was die Relevanz derartiger Angebote herausstellt.

Das Empfehlungsdokument „Anforderungen an die Hygiene bei der Reinigung und Desinfektion von Flächen“ von 2022 wurde vor allem in bestimmten Situationen oder bei konkreten Fragestellungen zu Rate gezogen. In GÄ wird die Empfehlung im beruflichen Alltag etwas regelmäßiger gelesen als auf der LE, was vermutlich auf die direktere Interaktion mit den Einrichtungen des Gesundheitswesens vor Ort zurückzuführen ist. Etwa die Hälfte aller Befragten schätzte die Empfehlung in der Sprache als relativ verständlich und im Aufbau als einigermaßen nachvollziehbar ein. Um zukünftig einerseits den Lesekomfort durch eine übersichtlichere Darstellung der konkreten Empfehlungen zu steigern und andererseits eine schnellere und leichtere Überarbeitung zu ermöglichen, hat die KRINKO im November 2023 mit einem Dokument zu SARS-CoV‑2 [8] erstmals eine Empfehlung in der neuen modularen Struktur herausgebracht. Zur neuen Struktur wurde eine Erläuterung veröffentlicht [8, 9].

Erwartungsgemäß war der informative Anhang nur etwa der Hälfte der Befragten bekannt, da dieser keinen Empfehlungscharakter besitzt und ausschließlich spezifische Hintergrundinformationen enthält, die eher für besonders Interessierte relevant sein könnten.

### Wissen über den Entstehungsprozess und die Evidenzkategorien der KRINKO-Empfehlungen

Wie bereits in der vorangegangenen Befragung zeigte sich auch in der aktuellen Befragung des ÖGD, dass der Entstehungsprozess der KRINKO-Empfehlungen der Fachöffentlichkeit nicht vollumfänglich bekannt ist. Insbesondere das öffentliche Stellungnahmeverfahren zu einem Empfehlungsentwurf mit Partizipation der Bundesländer und relevanter Fachverbände ist den Befragten aus den GÄ weniger geläufig, vermutlich weil sie hier nicht direkt partizipieren können. Das Verfahren stellt jedoch einen wichtigen Schritt in der Erstellung einer KRINKO-Empfehlung dar, da an dieser Stelle der Prozess für die LE transparent einsehbar ist und aktiv mitgestaltet werden kann, was wiederum die Qualität der Empfehlungen erhöht. Die fehlende Kenntnis des Verfahrens unterstreicht den Bedarf, die Arbeitsweise der KRINKO noch transparenter zu gestalten und adäquat zu kommunizieren.

Eine eingeschränkte Kenntnis wurde ebenso deutlich in Bezug auf die Kategorien zur Darstellung der Evidenzqualität, mit denen die Einzelempfehlungen versehen sind. Es war auffällig, dass die Befragten die Kategorien nicht nur als Indikator für die Evidenz wahrnahmen, sondern diese auch mit der Stärke einer Empfehlung gleichsetzten. Dies deckt sich mit den Ergebnissen der Pilotbefragung, bei der das Hygienefachpersonal die Kategorien auf ähnliche Weise interpretierte. Ebenso bestätigen die regelmäßig bei der KRINKO-Geschäftsstelle eingehenden individuellen Anfragen und Rückmeldungen aus der Fachöffentlichkeit diesen Eindruck. Dabei entfaltet auch eine mit den Worten „ohne Kategorie“ versehene Empfehlung die gleiche Vermutungswirkung wie ein Empfehlungswortlaut mit der Kategorie IA (d. h., die Empfehlung basiert auf gut konzipierten systematischen Reviews oder hochwertigen randomisierten kontrollierten Studien [4, 7]). Allen Zielgruppen dieser und der vorangegangenen Befragung war jedoch gemein, dass die Kategorien ihnen vor allem als Argumentationshilfe dienen, was die Relevanz der Empfehlungen auch für die berufliche Praxis der ÖGD-Mitarbeitenden verdeutlicht. In der vorliegenden Befragung war dieser Punkt auf KE etwas häufiger angegeben worden, was vermutlich wieder mit dem direkteren Austausch mit den Einrichtungen vor Ort begründbar ist.

Die KRINKO veröffentlichte zuletzt im Jahr 2010 die überarbeiteten Definitionen dieser Kategorien [7] und im Jahr 2012 eine Erläuterung der Arbeitsweise der KRINKO [10]. Um das Verständnis der Kategorien, aber auch die Transparenz im Entstehungsprozess einer KRINKO-Empfehlung zu fördern und zu stärken, erscheint es sinnvoll, weitere niederschwellige Erläuterungen für die Nutzenden anzubieten. Dazu könnten sich z. B. kurze Musterpräsentationen zum Entstehungsprozess bzw. zu den Kategorien eignen, um diese leicht und schnell zu visualisieren. Auch eine regelmäßige Durchführung von Webseminaren wäre denkbar, setzt jedoch ausreichende personelle Kapazitäten z. B. in der KRINKO-Geschäftsstelle voraus.

### Formate für die Verbreitung aktueller Informationen zu KRINKO-Inhalten

In welchem Ausmaß Mitarbeitende des ÖGD tatsächlich Social-Media-Kanäle, Onlineplattformen oder Apps zu Informationszwecken heranziehen, wurde in der hier vorgestellten Befragung zwar nicht direkt erfasst, jedoch wurde deutlich, dass die Teilnehmenden zumindest seltener über diese digitalen Angebote von neuen KRINKO-Empfehlungen erfahren. Ebenso spielten Social-Media-Kanäle und „Agora“ (eine Kollaborationsplattform für den ÖGD [11], die sich zum Befragungszeitpunkt in Umstrukturierung befand) sowie die ÖGD-News-App der Akademie für Öffentliches Gesundheitswesen (AÖGW) eine sehr untergeordnete Rolle bei der Einholung von Informationen zur vorliegenden Befragung. Dies kann möglicherweise daran liegen, dass diese Kanäle bisher seitens der KRINKO bzw. Geschäftsstelle kaum zur Verbreitung von Informationen genutzt werden. Ein verstärkter Einsatz innovativer digitaler Kommunikationskanäle könnte die Reichweite von Informationen über die KRINKO erhöhen, die Adressatengruppen gezielter ansprechen und Beiträge niederschwelliger und nutzerfreundlicher zugänglich machen. Eine Stärkung der digitalen Präsenz erscheint daher sinnvoll.

## Fazit

Die vorliegende Befragung liefert wichtige Erkenntnisse zur Perzeption der KRINKO-Empfehlungen im ÖGD im Bereich Infektionshygiene. Wie auch bei der Pilotbefragung des Hygienefachpersonals medizinischer Einrichtungen zeigt sich, dass Onlinebefragungen ein geeignetes Instrument darstellen, um ein Meinungsbild zur Perzeption der KRINKO-Empfehlungen zu skizzieren. Die aktuelle Befragung der Mitarbeitenden des ÖGD verdeutlicht, dass die Abfrage der Bedarfe und Wahrnehmungen eine gute Basis ist, bestehende Angebote auf eine anwendungsfreundlichere und zielgruppengerechtere Kommunikation weiterzuentwickeln. Künftige Befragungen könnten vertiefend auf zielgruppenspezifische Belange eingehen, beispielsweise durch erweiterte Befragungsdesigns mit differenzierten Auswahloptionen (z. B. in Form von Drop-down-Listen).

Die Ergebnisse der aktuellen Befragung werden umfassend an die KRINKO zurückgespiegelt, damit sie in die Erstellung zukünftiger Empfehlungen sowie in die Darstellung der Arbeitsweise der Kommission einfließen können.

## Supplementary Information

Originalfragebogen zur Analyse der Perzeption der KRINKO-Empfehlungen im ÖGD

Zusätzliche Abbildungen und Tabellen
