# Supplementary material for: How are the recommendations of the Commission for Infection Prevention and Hygiene in Healthcare and Nursing (KRINKO) perceived in the public health service in Germany? Report on the results of an online survey of public health service staff conducted by the Robert Koch Institute (RKI)
Source: Bundesgesundheitsblatt Gesundheitsforschung Gesundheitsschutz. 2025 Oct 21;69(1):108–16. [Article in German] doi: 10.1007/s00103-025-04131-4 (PMC12764681; doi:10.1007/s00103-025-04131-4)
Supplement: Supplementary file 1 — Originalfragebogen zur Analyse der Perzeption der KRINKO-Empfehlungen im ÖGD [file 103_2025_4131_MOESM1_ESM.pdf]

Onlinematerial 1 zum Bericht über die Ergebnisse einer Onlinebefragung von Mitarbeitenden des ÖGD durch das RKI „Wie werden die KRINKO-Empfehlungen im Öffentlichen Gesundheitsdienst (ÖGD) wahrgenommen?“

Fragebogen zur Analyse der Perzeption der KRINKO-Empfehlungen II – Fokus ÖGD

Herzlich willkommen!

Über diese Befragung

In der diesjährigen Befragung möchten wir wissen, wie und in welchem Umfang die Empfehlungen der Kommission für Krankenhaushygiene und Infektionsprävention (KRINKO) bei Personen wahrgenommen werden, die im öffentlichen Gesundheitsdienst tätig sind und die sich in ihrer Tätigkeit direkt oder indirekt mit den Empfehlungen der KRINKO befassen.

Die Befragung wird durchgeführt vom **Fachgebiet 14 „Angewandte Infektions- und Krankenhaushygiene“ des Robert Koch-Instituts**. Das Fachgebiet 14 ist das wissenschaftliche Sekretariat und die Geschäftsstelle der KRINKO. Die Fachgebietsleitung liegt bei Prof. Dr. Mardjan Arvand.

Die primäre Ansprechpartnerin für die Befragung ist Melanie Winkler.

Kontakt:
Robert Koch-Institut
Fachgebiet 14 „Angewandte Infektions- und Krankenhaushygiene“
Nordufer 20
13353 Berlin
KRINKO-Perzeption@rki.de

- Die Befragung wird **anonym** durchgeführt und wurde mit der Datenschutzbeauftragten des RKI abgestimmt. Anonym bedeutet, dass anhand Ihrer Angaben keinerlei Rückschlüsse auf Ihre Identität möglich sind. Die Teilnahme ist freiwillig.
- Die Teilnahme an der Befragung dauert **circa 10 Minuten**.
- Sie können die Befragung jederzeit abbrechen oder einzelne Fragen unbeantwortet lassen.
- Die Ergebnisse werden uns helfen, die Kommunikation der Empfehlungen zu verbessern und spezifische Bedarfe verschiedener Anwendergruppen gezielter zu adressieren.
- Es ist geplant, die Ergebnisse der Befragung, z. B. im Epidemiologischen Bulletin, zu veröffentlichen.

Vielen Dank, dass Sie sich die Zeit für unsere Befragung nehmen!

[Befragung jetzt starten]

[Ich möchte nicht teilnehmen.]

BLOCK I - Über die Befragten

1. Ich bin tätig in einer/einem

O kommunalen Gesundheitsamt

- Gesundheitsamt – Amtsleitung
- Gesundheitsamt – Ärzt:in mit Aufgaben im Bereich Hygieneüberwachung/Infektionsschutz
- Gesundheitsamt – Hygieneinspektor:in / Hygienekontrolleur:in / Gesundheitsaufseher:in
- Gesundheitsamt – andere nichtärztliche Funktion
- Gesundheitsamt – andere ärztliche Funktion

O Landesgesundheitsbehörde/-ministerium

- Landesgesundheitsbehörde/-ministerium – Bereich Hygiene/Infektionsschutz
- Landesgesundheitsbehörde/-ministerium – Bereich Medizinprodukte
- Landesgesundheitsbehörde/-ministerium – anderer Bereich

O Bundesoberbehörde/-ministerium für Gesundheit

- Bundesoberbehörde/-ministerium für Gesundheit – Bereich Hygiene/Infektionsschutz
- Bundesoberbehörde/-ministerium für Gesundheit – anderer Bereich

O anderen Einrichtung

2. In welchem Bundesland sind Sie tätig?

Scrollleiste mit Auflistung aller Bundesländer und zusätzlicher Option „keine Angabe".

3. Welche der folgenden Tätigkeiten und Aufgaben gehören zu den Schwerpunkten Ihrer Arbeit? (Mehrfachnennungen möglich)

O Beratungs- und Überwachungsaufgaben im Bereich der Hygiene

- Einrichtungen nach § 23 (3) IfSG (Krankenhäuser, andere medizinische Einrichtungen und Einrichtungen der Pflege)
- Gemeinschaftseinrichtungen nach § 33 IfSG (Einrichtungen, in denen überwiegend minderjährige Personen betreut werden)
- Einrichtungen nach § 35 IfSG (Unternehmen der Pflege und Eingliederungshilfe)
- Einrichtungen nach § 36 IfSG (Infektionsschutz bei bestimmten Einrichtungen, Unternehmen und Personen)
- Einrichtungen nach § 37 IfSG (Beschaffenheit von Wasser für den menschlichen Gebrauch sowie von Wasser zum Schwimmen oder Baden in Becken oder Teichen)

O Netzwerkarbeit (z. B. MRE-Netzwerke)

O Netzwerkarbeit (z. B. MRE-Netzwerke), Moderation

O Beratungs- und Überwachungsaufgaben im Bereich Aufbereitung der Medizinprodukte

O Gesundheitsberichterstattung, Epidemiologie

O Bearbeitung von meldepflichtigen Erregern

O andere Tätigkeiten

BLOCK II - KRINKO allgemein

4. Wie gut kennen Sie die für Ihre Tätigkeit relevanten Empfehlungen der KRINKO? (Selbsteinschätzung)

0 = gar nicht 10 = sehr gut

0 5 10

O O O O O O O O O O O

5. Wie häufig lesen Sie in den Originaltexten der KRINKO-Empfehlungen nach?

O mehrmals pro Woche

O mehrmals im Monat

O unregelmäßig, kann ich nicht benennen

O anlassbezogen

O nie

6. Falls Frage 5 zutreffend: In welcher Form lesen Sie die Originaltexte der Empfehlungen bevorzugt?

O in der Loseblattsammlung (Printfassung in den grünen Ordnern vom Elsevier-Verlag)

O im Bundesgesundheitsblatt (d.h. in der Druckausgabe der Zeitschrift)

O ausgedrucktes PDF

O elektronisch / auf dem Bildschirm (d.h. online oder als gespeicherte Datei)

7. Auf welchen Wegen erfahren Sie von neuen KRINKO-Empfehlungen?

(Mehrfachnennungen möglich)

O Bundesgesundheitsblatt

O Homepage des RKI

O RKI-Newsletter

O Social Media-Kanäle des RKI (z. B. X, LinkedIn)

O andere Social Media-Kanäle

O AGORA

O ÖGD-News-App der AÖGW

O Informationen der Fachgesellschaften

O Intranet meiner Einrichtung

O Fachzeitschriften

O Kongresse, z. B. Vorträge

O Kolleg:innen

O andere

8. Woher kennen Sie die Inhalte der KRINKO-Empfehlungen? (Mehrfachnennungen möglich)

O aus den Originaldokumenten

O aus den Musterpräsentationen des RKI

O aus Informationen/Fortbildungen/Schulungen/Checklisten in meiner Einrichtung

O aus externen Fortbildungen

O von Kolleg:innen

O aus der Ausbildung/Studium

O Ich kenne die Inhalte der KRINKO-Empfehlungen nicht.

9. Wenn ich eine Verständnisfrage zu konkreten Aussagen einer KRINKO-Empfehlung habe, …

O frage ich Kolleg:innen innerhalb meiner Einrichtung.

O frage ich Kolleg:innen außerhalb meiner Einrichtung.

O schreibe ich an Infektionshygiene@rki.de.

O schaue ich auf die RKI-Homepage.

O mache ich eine freie Internetsuche zu dem Thema.

O mache ich etwas anderes als hier erwähnt.

10. Vermitteln Sie im Rahmen Ihrer Tätigkeit auch Inhalte von KRINKO-Empfehlungen, z. B. im Rahmen von Fortbildungen, Schulungen, Gesprächen, etc.?

O ja

O eher indirekt, z. B. in Begehungsberichten

O nein

10.

a. Wie vermitteln Sie diese Inhalte? (Mehrfachnennungen möglich)

O Vorträge

O Workshops

O ich erstelle Materialien z. B. Flyer, Informationsschreiben etc.

O individuelle Beratungen, z. B. am Telefon

O individuelle Gespräche bei Begehungen

O indirekt in Begehungsberichten

O anderes

b. Für die Kommunikation/Vermittlung der Inhalte der KRINKO-Empfehlungen… (Mehrfachnennungen möglich)

O nutze ich Originaltexte (in Auszügen, z. B. zur Veranschaulichung).

O nutze ich existierende Materialien, z. B. die Musterpräsentationen des RKI.

O nutze ich andere Materialien, z. B. Checklisten.

O stelle ich selbst Materialien zusammen, die ich auf die jeweilige Einrichtung bzw. den Bereich zuschneide.

O Ich kommuniziere die Inhalte der KRINKO-Empfehlungen nicht explizit als solche.

11. Welche Aspekte aus dem Entstehungsprozess einer KRINKO-Empfehlung sind Ihnen bekannt? (Mehrfachnennungen möglich)

O Zunächst wird ein Empfehlungsentwurf in einer Arbeitsgruppe der KRINKO erarbeitet. Der Entwurf wird anschließend von der gesamten Kommission durchgesehen, überarbeitet und abgestimmt.

O Der so entstandene Empfehlungsentwurf wird anschließend in ein fachliches Stellungnahmeverfahren (Anhörung) an die zuständigen Fachgesellschaften und Behörden sowie Institutionen auf Länder- und Bundesebene versendet.

O Unter Berücksichtigung und Prüfung der Rückmeldungen aus der Anhörung wird der Entwurf nochmals in der KRINKO beraten, verabschiedet und veröffentlicht.

O Ich bin mit dem Entstehungsprozess der KRINKO-Empfehlungen nicht vertraut.

12. Welche Aussagen treffen für Sie in Bezug auf die Kategorien der KRINKO-Empfehlungen (nach dem Stand von 2010) zu? (Mehrfachnennungen möglich)

Tab. 2 Kategorien in der Richtlinie für Krankenhaushygiene und Infektionsprävention (2010)
Kategorie IA:<br>Diese Empfehlung basiert auf gut konzipierten systematischen Reviews oder einzelnen hochwertigen randomisierten kontrollierten Studien.
Kategorie IB:<br>Diese Empfehlung basiert auf klinischen oder hochwertigen epidemiologischen Studien *und* strengen, plausiblen und nachvollziehbaren theoretischen Ableitungen.
Kategorie II:<br>Diese Empfehlung basiert auf hinweisenden Studien/Untersuchungen *und* strengen, plausiblen und nachvollziehbaren theoretischen Ableitungen.
Kategorie III:<br>Maßnahmen, über deren Wirksamkeit nur unzureichende oder widersprüchliche Hinweise vorliegen, deshalb ist eine Empfehlung nicht möglich.
Kategorie IV:<br>Anforderungen, Maßnahmen und Verfahrensweisen, die durch allgemein geltende Rechtsvorschriften zu beachten sind.

O Die Kategorien der KRINKO-Empfehlungen nach dem Stand von 2010 nehme ich als Ausdruck der zugrundeliegenden Evidenz wahr.

O Die Kategorien der KRINKO-Empfehlungen nach dem Stand von 2010 nehme ich als Ausdruck der Stärke einer Empfehlung wahr.

O Die Kategorie einer Empfehlung hilft mir, in bestimmten Situationen Entscheidungen zu treffen oder zu argumentieren.

O Für meinen Berufsalltag spielen die Kategorien der Empfehlungen keine besondere Rolle.

BLOCK III – Spezieller Teil

Im folgenden Teil der Befragung befassen wir uns spezifisch mit dem Empfehlungsdokument „Anforderungen an die Hygiene bei der Reinigung und Desinfektion von Flächen“ von 2022.

13. **Ist Ihnen die aktuelle KRINKO-Empfehlung „Anforderungen an die Hygiene bei der Reinigung und Desinfektion von Flächen“ von 2022 inhaltlich vertraut?**

O ja

O teilweise vertraut

O nein

14. **In welchen Situationen lesen Sie im Originaltext der aktuellen KRINKO-Empfehlung „Anforderungen an die Hygiene bei der Reinigung und Desinfektion von Flächen“ von 2022 nach?**

O in meinem Berufsalltag regelmäßig

O nur in besonderen Situationen / bei ungelösten Fragen / Problemen

O Ich lese nicht den Originaltext, der Inhalt ist mir aber aus anderen Materialien, z. B. Lehrbüchern, Fortbildungen zum Thema etc., vertraut.

O gar nicht

15.

a. **Welchen der folgenden Sätze stimmen Sie hinsichtlich der aktuellen Empfehlung „Anforderungen an die Hygiene bei der Reinigung und Desinfektion von Flächen“ von 2022 zu?**

O Die **Sprache/Formulierungen** in der aktuellen KRINKO-Empfehlung „**Anforderungen an die Hygiene bei der Reinigung und Desinfektion von Flächen**“ finde ich im Großen und Ganzen *klar und verständlich*.

O Die **Sprache/Formulierungen** in der aktuellen KRINKO-Empfehlung „**Anforderungen an die Hygiene bei der Reinigung und Desinfektion von Flächen**“ finde ich im Großen und Ganzen *relativ verständlich*.

O Die **Sprache/Formulierungen** in der aktuellen KRINKO-Empfehlung „**Anforderungen an die Hygiene bei der Reinigung und Desinfektion von Flächen**“ finde ich im Großen und Ganzen *zu kompliziert*.

b. **Welchen der folgenden Sätze stimmen Sie hinsichtlich der aktuellen Empfehlung „Anforderungen an die Hygiene bei der Reinigung und Desinfektion von Flächen“ von 2022 zu?**

O Der **Aufbau und die Struktur** der aktuellen KRINKO-Empfehlung „**Anforderungen an die Hygiene bei der Reinigung und Desinfektion von Flächen**“ empfinde ich als *leicht* nachvollziehbar.

O Der **Aufbau und die Struktur** der aktuellen KRINKO-Empfehlung „**Anforderungen an die Hygiene bei der Reinigung und Desinfektion von Flächen**“ empfinde ich als *einigermaßen* nachvollziehbar.

O Der **Aufbau und die Struktur** der aktuellen KRINKO-Empfehlung „**Anforderungen an die Hygiene bei der Reinigung und Desinfektion von Flächen**“ empfinde ich als *schwer* nachvollziehbar.

Im "Informativen Anhang der Empfehlung „Anforderungen an die Hygiene bei der Reinigung und Desinfektion von Flächen“ wurden Angaben zur Wiederanzüchtbarkeit von Mikroorganismen und Viren auf Flächen zusammengestellt, um Interessierten weiterführende Informationen zur Verfügung zu stellen. Weiterhin werden Hinweise zu Methoden zur Bewertung der Ergebnisqualität der Reinigung bzw. desinfizierenden Flächenreinigung gegeben.

16. **Ist Ihnen der informative Anhang der Empfehlung „Anforderungen an die Hygiene bei der Reinigung und Desinfektion von Flächen“ von 2022 bekannt?**

    O Ja, mir ist der informative Anhang bekannt.

    O Ja, mir ist der informative Anhang bekannt und er ist für meine Tätigkeit relevant.

    O Ja, mir ist der informative Anhang bekannt, aber er ist für meine Tätigkeit nicht relevant.

    O Nein, mir ist der informative Anhang nicht bekannt.

BLOCK IV - Abschluss

Würden Sie uns abschließend mitteilen, wie Sie von dieser Befragung erfahren haben?

O Anzeige in Hygiene & Medizin

O Anzeige im Epidemiologischen Bulletin

O Homepage des RKI

O Hinweis von Kolleg:innen

O Social Media

O ÖGD-News-App der AÖGW

O AGORA

O anderes

Herzlichen Dank für Ihre Teilnahme und Ihre Zeit! Ihre Rückmeldung ist für uns sehr hilfreich!

Die Befragung ist hiermit beendet.

[Zurück]

[Abschließen]
