# Supplementary material for: How are the recommendations of the Commission for Infection Prevention and Hygiene in Healthcare and Nursing (KRINKO) perceived in the public health service in Germany? Report on the results of an online survey of public health service staff conducted by the Robert Koch Institute (RKI)
Source: Bundesgesundheitsblatt Gesundheitsforschung Gesundheitsschutz. 2025 Oct 21;69(1):108–16. [Article in German] doi: 10.1007/s00103-025-04131-4 (PMC12764681; doi:10.1007/s00103-025-04131-4)
Supplement: Supplementary file 2 — Zusätzliche Abbildungen und Tabellen [file 103_2025_4131_MOESM2_ESM.pdf]

Onlinematerial 2 zum Bericht über die Ergebnisse einer Onlinebefragung von Mitarbeitenden des ÖGD durch das RKI „Wie werden die KRINKO-Empfehlungen im Öffentlichen Gesundheitsdienst (ÖGD) wahrgenommen?“

Antworten zu der Frage „Welche der folgenden Tätigkeiten und Aufgaben gehören zu den Schwerpunkten Ihrer Arbeit?“.

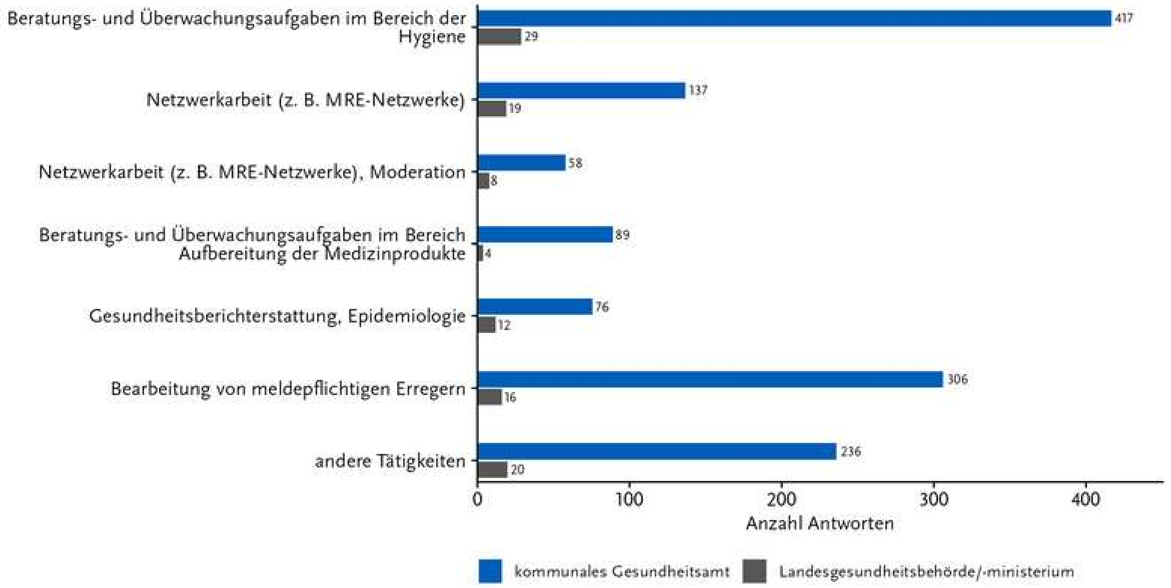

Abb. A1 | Anzahl Antworten: 526; davon GA: 481 und LE: 45. Mehrfachnennungen waren möglich. Onlinebefragung zur Wahrnehmung der KRINKO-Empfehlungen im ÖGD 11-12/2023

Antworten zu der Frage „Wie gut kennen Sie die für Ihre Tätigkeit relevanten Empfehlungen der KRINKO?“.

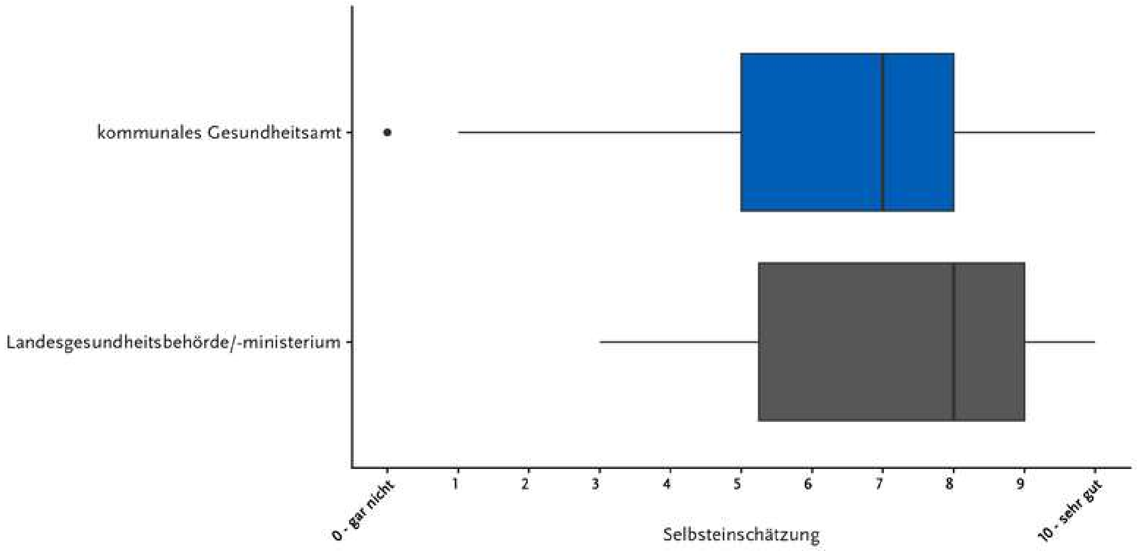

Abb. A2 | Anzahl Antworten: 513; davon GA: 471 und LE: 42. Onlinebefragung zur Wahrnehmung der KRINKO-Empfehlungen im ÖGD 11-12/2023

Antworten zu der Frage „Wie häufig lesen Sie in den Originaltexten der KRINKO-Empfehlungen nach?“.

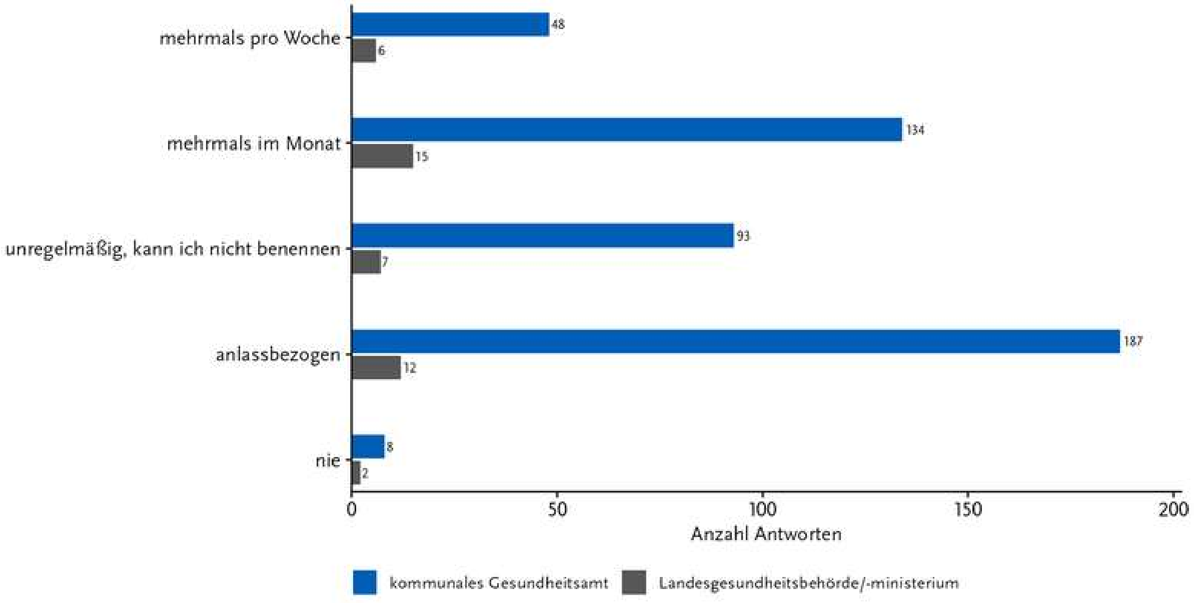

Abb. A3 | Anzahl Antworten: 512; davon GA: 470 und LE: 42. Onlinebefragung zur Wahrnehmung der KRINKO-Empfehlungen im ÖGD 11-12/2023

Antworten zu der Frage „Vermitteln Sie im Rahmen Ihrer Tätigkeit auch Inhalte von KRINKO-Empfehlungen, z. B. im Rahmen von Fortbildungen, Schulungen, Gesprächen etc.?“.

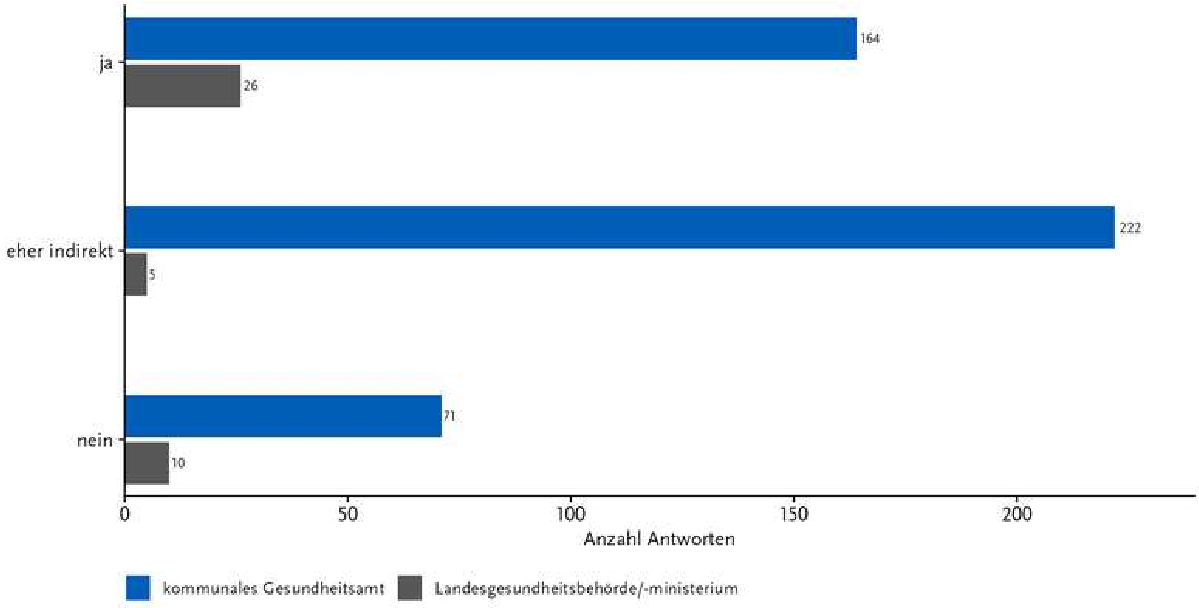

Abb. A4 | Anzahl Antworten: 498; davon GA: 457 und LE: 41. Onlinebefragung zur Wahrnehmung der KRINKO-Empfehlungen im ÖGD 11-12/2023

Antworten zu der Frage „Ist Ihnen die aktuelle KRINKO-Empfehlung „Anforderungen an die Hygiene bei der Reinigung und Desinfektion von Flächen“ von 2022 inhaltlich vertraut?“.

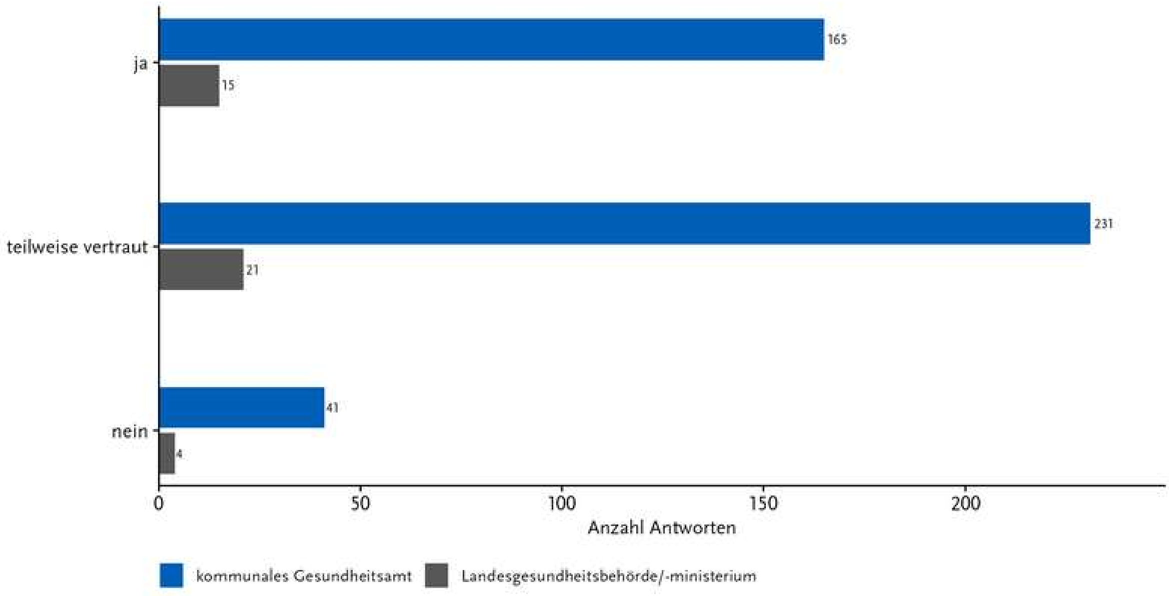

Abb. A5 | Anzahl Antworten: 477; davon GA: 437 und LE: 40. Onlinebefragung zur Wahrnehmung der KRINKO-Empfehlungen im ÖGD 11-12/2023

Antworten zu der Frage „In welchen Situationen lesen Sie im Originaltext der aktuellen KRINKO-Empfehlung „Anforderungen an die Hygiene bei der Reinigung und Desinfektion von Flächen“ von 2022 nach?“.

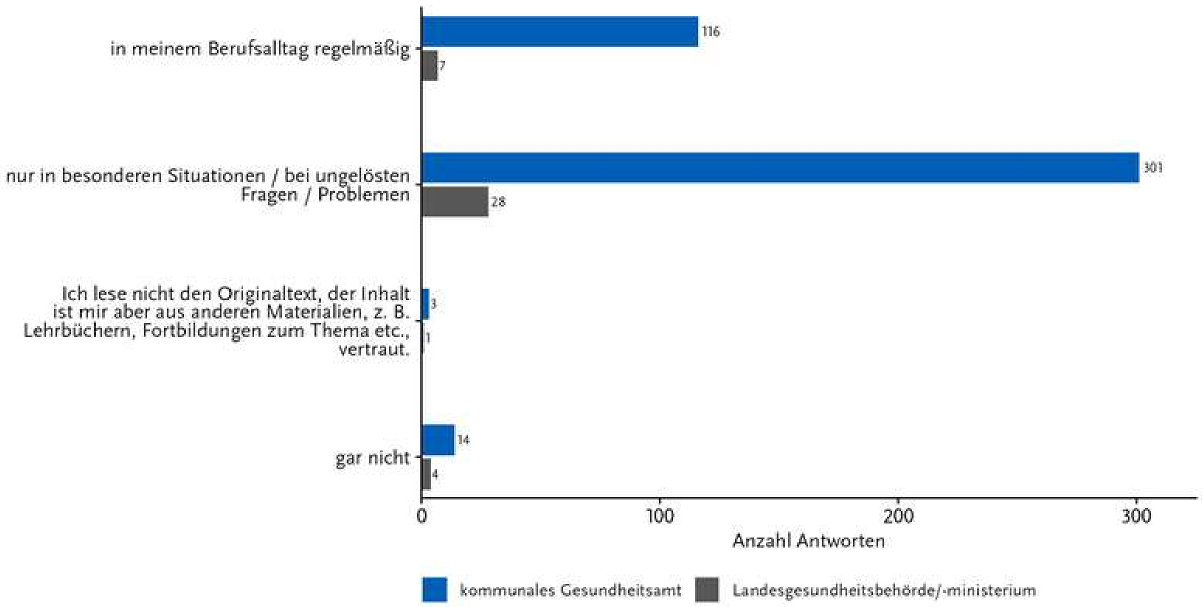

Abb. A6 | Anzahl Antworten: 474; davon GA: 434 und LE: 40. Onlinebefragung zur Wahrnehmung der KRINKO-Empfehlungen im ÖGD 11-12/2023

Antworten zu der Frage „Welchen der folgenden Sätze *[bzgl. Sprache und Formulierungen]* stimmen Sie hinsichtlich der aktuellen KRINKO-Empfehlung „Anforderungen an die Hygiene bei der Reinigung und Desinfektion von Flächen“ von 2022 zu?“ - „Die Sprache/Formulierungen in der aktuellen KRINKO-Empfehlung „Anforderungen an die Hygiene bei der Reinigung und Desinfektion von Flächen“ finde ich im Großen und Ganzen…“.

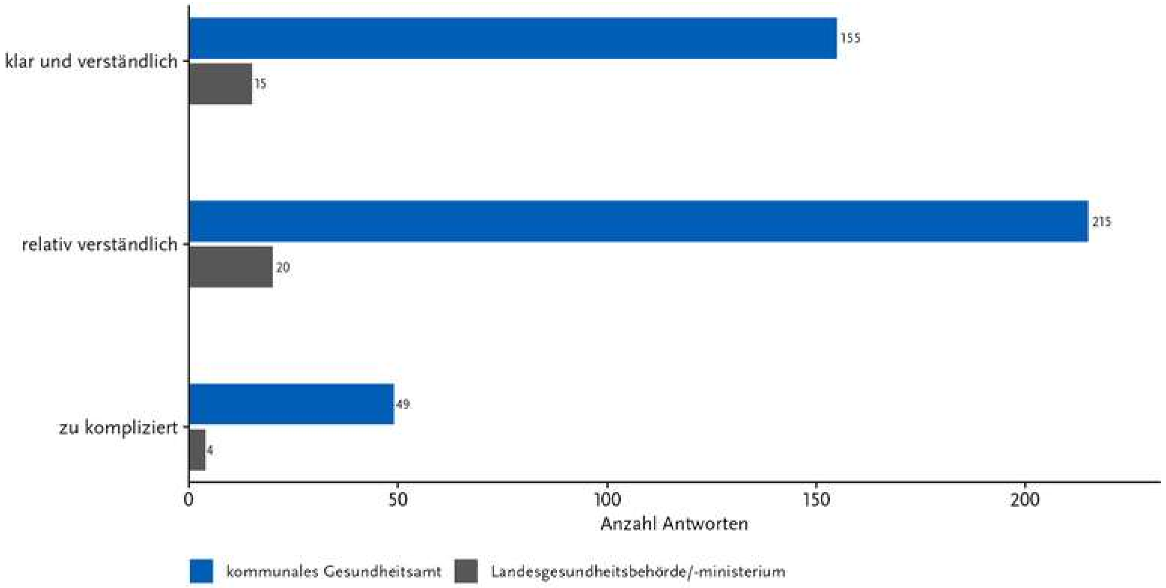

Abb. A7 | Anzahl Antworten: 458; davon GA: 419 und LE: 39. Onlinebefragung zur Wahrnehmung der KRINKO-Empfehlungen im ÖGD 11-12/2023

Antworten zu der Frage „Welchen der folgenden Sätze *[bzgl. Aufbau und Struktur]* stimmen Sie hinsichtlich der aktuellen KRINKO-Empfehlung „Anforderungen an die Hygiene bei der Reinigung und Desinfektion von Flächen“ von 2022 zu?“ - „Den Aufbau und die Struktur der aktuellen KRINKO-Empfehlung „Anforderungen an die Hygiene bei der Reinigung und Desinfektion von Flächen“ empfinde ich als…“.

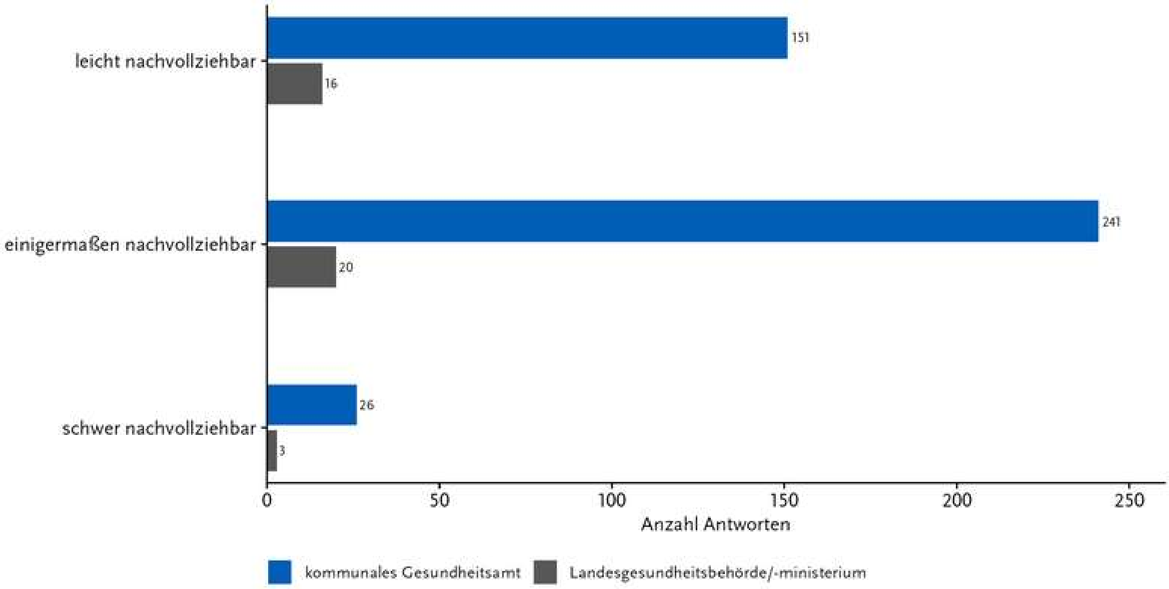

Abb. A8 | Anzahl Antworten: 457; davon GA: 418 und LE: 39. Onlinebefragung zur Wahrnehmung der KRINKO-Empfehlungen im ÖGD 11-12/2023

Onlinematerial 2

Antworten zu der Frage „Ist Ihnen der informative Anhang der Empfehlung „Anforderungen an die Hygiene bei der Reinigung und Desinfektion von Flächen“ von 2022 bekannt?“.

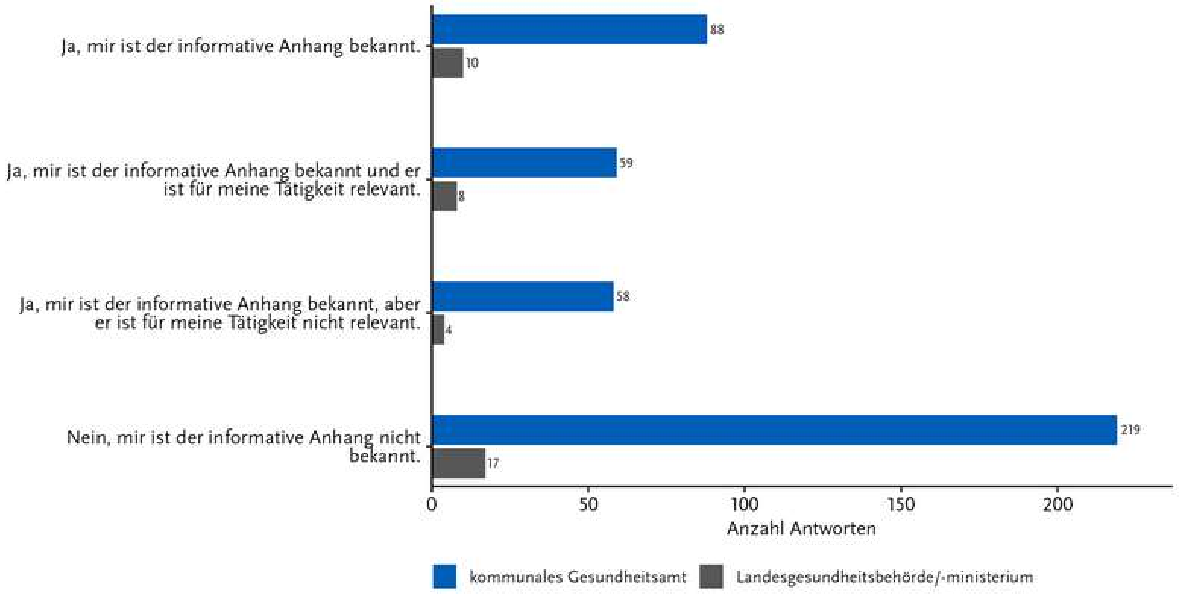

Abb. A9 | Anzahl Antworten: 463; davon GA: 424 und LE: 39. Onlinebefragung zur Wahrnehmung der KRINKO-Empfehlungen im ÖGD 11-12/2023

Tab. A1 | Antworten zu der Frage „Woher kennen Sie die Inhalte der KRINKO-Empfehlungen?“. Mehrfachnennungen waren möglich. Onlinebefragung zur Wahrnehmung der KRINKO-Empfehlungen im ÖGD 11-12/2023

**Antworten insgesamt: 500**	**GA (n=459)**	**LE (n=41)**
aus den Originaldokumenten	72,1 %	82,9 %
aus den Musterpräsentationen des RKI	29,0 %	29,3 %
aus Informationen/Fortbildungen/Schulungen/Checklisten in meiner Einrichtung	32,7 %	14,6 %
aus externen Fortbildungen	41,4 %	29,3 %
von Kolleg:innen	36,4 %	19,5 %
aus der Ausbildung/Studium	27,0 %	12,2 %
Ich kenne die Inhalte der KRINKO-Empfehlungen nicht.	1,1 %	0,0 %

Tab. A2 | Antworten zu der Frage „Wenn ich eine Verständnisfrage zu konkreten Aussagen einer KRINKO-Empfehlung habe …“. Mehrfachnennungen waren möglich. Onlinebefragung zur Wahrnehmung der KRINKO-Empfehlungen im ÖGD 11-12/2023

**Antworten insgesamt: 498**	**GA (n=457)**	**LE (n=41)**
frage ich Kolleg:innen innerhalb meiner Einrichtung.	80,7 %	75,6 %
frage ich Kolleg:innen außerhalb meiner Einrichtung.	35,7 %	41,5 %
schreibe ich an Infektionshygiene@rki.de.	4,4 %	19,5 %
schaue ich auf die RKI-Homepage.	65,4 %	51,2 %
mache ich eine freie Internetsuche zu dem Thema.	58,0 %	53,7 %
mache ich etwas anderes als hier erwähnt.	4,8 %	9,8 %

**Tab. A3 | Antworten zu der Frage „Wie vermitteln Sie diese Inhalte?“.** Mehrfachnennungen waren möglich. Diese Frage war nur an die Teilnehmenden gerichtet, welche die vorherige Frage (siehe Abb. 4 im Onlinematerial) mit „ja“ beantwortet haben. Onlinebefragung zur Wahrnehmung der KRINKO-Empfehlungen im ÖGD 11-12/2023		
**Antworten insgesamt: 417**	**GA (n=386)**	**LE (n=31)**
Vorträge	21,0 %	54,8 %
Workshops	2,3 %	22,6 %
ich erstelle Materialien, z. B. Flyer, Informationsschreiben etc.	16,6 %	41,9 %
individuelle Beratungen, z. B. am Telefon	75,4 %	74,2 %
individuelle Gespräche bei Begehungen	88,9 %	58,1 %
indirekt in Begehungsberichten	83,4 %	45,2 %
anderes	4,2 %	9,7 %

**Tab. A4 | Antworten zu der Frage „Für die Kommunikation/Vermittlung der Inhalte der KRINKO-Empfehlungen… “.** Mehrfachnennungen waren möglich. Diese Frage war nur an die Teilnehmenden gerichtet, welche die vorherige Frage (siehe Abb. 4 im Onlinematerial) mit „ja“ oder „eher indirekt“ beantwortet haben. Onlinebefragung zur Wahrnehmung der KRINKO-Empfehlungen im ÖGD 11-12/2023		
**Antworten insgesamt: 412**	**GA (n=381)**	**LE (n=31)**
nutze ich Originaltexte (in Auszügen, z. B. zur Veranschaulichung).	79,5 %	90,3 %
nutze ich existierende Materialien, z. B. die Musterpräsentationen des RKI.	29,4 %	41,9 %
nutze ich andere Materialien, z. B. Checklisten.	46,2 %	41,9 %
stelle ich selbst Materialien zusammen, die ich auf die jeweilige Einrichtung bzw. den Bereich zuschneide.	45,1 %	48,4 %
Ich kommuniziere die Inhalte der KRINKO-Empfehlungen nicht explizit als solche.	3,9 %	0,0 %

**Tab. A5 | Antworten zu der Abschlussfrage „Würden Sie uns abschließend mitteilen, wie Sie von dieser Befragung erfahren haben?“.** Mehrfachnennungen möglich. Onlinebefragung zur Wahrnehmung der KRINKO-Empfehlungen im ÖGD 11-12/2023		
**Antworten insgesamt: 465**	**GA (n=426)**	**LE (n=39)**
Anzeige in *Hygiene & Medizin*	3,5 %	10,3 %
Anzeige im *Epidemiologischen Bulletin*	28,4 %	23,1 %
Homepage des RKI	15,7 %	28,2 %
Hinweis von Kolleg:innen	37,1 %	43,6 %
Social Media	0,0 %	2,6 %
ÖGD-News-App der AÖGW	4,5 %	5,1 %
AGORA	0,9 %	0,0 %
anderes	23,7 %	12,8 %
